# P-684. Wheezing, Fever, Snot, Oh My! Comparing Respiratory Syncytial Virus (RSV) and Human Metapneumovirus (HMPV) in Adults: Implications for Vaccine Development

**DOI:** 10.1093/ofid/ofae631.880

**Published:** 2025-01-29

**Authors:** Colin Samoriski, Ann R Falsey, Angela R Branche, Daniel P Croft, Edward E Walsh

**Affiliations:** University of Rochester, Rochester, New York; University of Rochester School of Medicine, Rochester, New York; University of Rochester, Rochester, New York; University of Rochester Medical Center, Rochester, New York; University of Rochester, Rochester, New York

## Abstract

**Background:**

RSV and HMPV cause acute respiratory infections (ARI) in children and adults. Two RSV vaccines for older adults are now licensed, and HMPV vaccines are in development. Limited data exist comparing clinical disease and impact of HMPV to RSV in older adults.

**Table 1.**
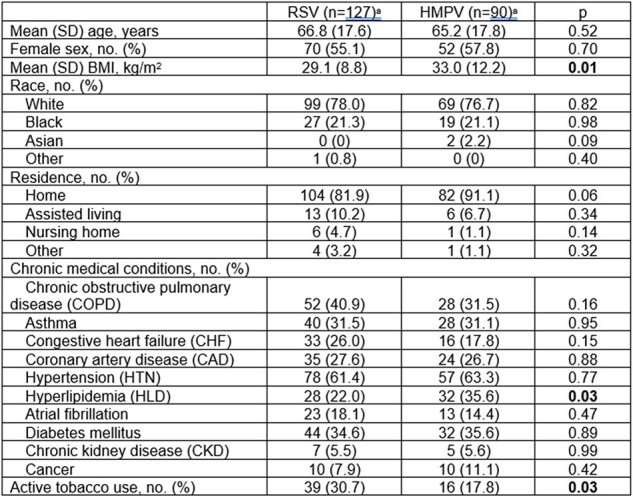
Demographic and Baseline Characteristics

**Methods:**

We analyzed data from two studies of prospectively enrolled hospitalized adults with ARI from 2008-2012 and 2018-2023 and compared demographics, clinical characteristics and outcomes of HMPV and RSV. Similar inclusion/exclusion criteria and RT-PCR diagnosis were used for both studies, with addition of serology only in the first. All cases were adjudicated for bacterial coinfection and radiographic pneumonia by 1 pulmonary and 3 ID physicians. Comparisons used t-test or χ^2^.

**Table 2.**
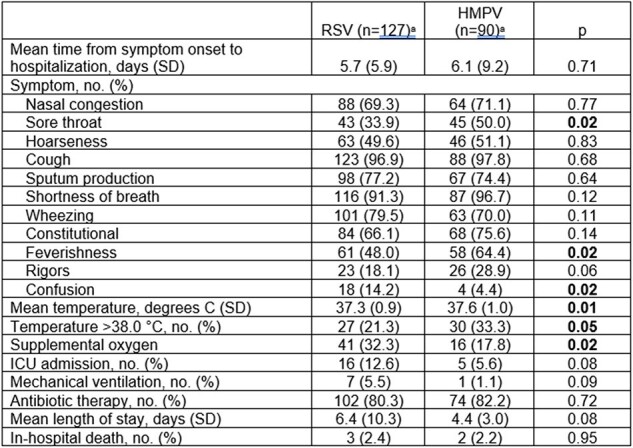
Signs and Symptoms at Admission, Clinical Characteristics, and Outcomes

**Results:**

Of 2,012 patients enrolled, 127 (6.3%) and 90 (4.5%) had RSV or HMPV, respectively: 5 patients with RSV/HMPV co-infection were excluded from analysis. Mean age (67 years), sex, race and ethnicity were similar (Table 1). HMPV patients were less likely to reside in congregate settings (7.8 v 14.9%, p=0.14) or have COPD (31.5 v 40.9%, p=0.16), CHF (17.8 v 26.0%, p=0.15), or active tobacco use (17.8 v 30.7%, p=0.03). Clinical presentation also differed with HMPV more associated with sore throat (50.0 v 33.9%, p=0.02), feverishness (64.4 v 48.0%, p=0.02), temperature >38^o^C (33.3 v 21.3%, p=0.05), and constitutional symptoms (75.6 v 66.1%, p=0.14). (Table 2) HMPV patients required less ICU care (5.6 v 12.6%, p=0.08) and mechanical ventilation (1.1 v 5.5%, p=0.09). Bacterial coinfection occurred equally, but HMPV patients judged as viral infection alone were more often adjudicated as pneumonia (35.6 v 22.8%, p=0.006). (Fig. 1).

**Figure 1.**
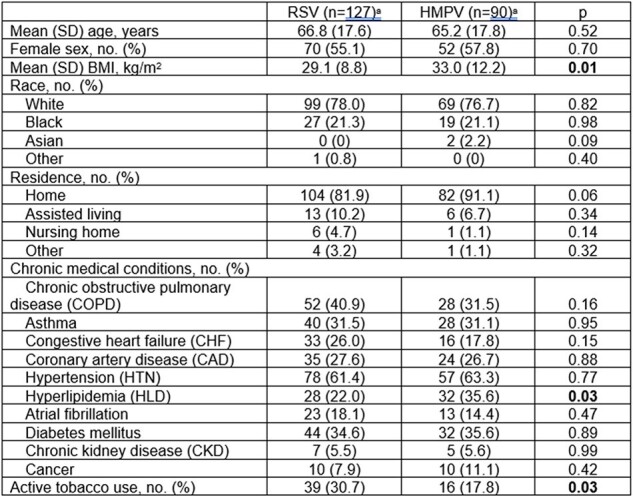
Adjudicated Radiographic Pneumonia with Microbiologic Diagnosis

**Conclusion:**

Although RSV and HMPV are closely related viruses, we found differences in the clinical features of adults hospitalized with ARI. While HMPV accounted for about 3/4 the number of cases compared to RSV, HPMV patients were generally healthier and community-dwelling with fewer high-risk cardiopulmonary conditions. Notably, HMPV patients presented with more flu-like symptoms and were judged more often to have viral pneumonia. These results support development of an adult HMPV vaccine. Ongoing gene expression analyses may elucidate mechanisms underlying these differences.

**Disclosures:**

**Ann R. Falsey, MD**, ADMA: Board Member|BioFire Diagnostics: Grant/Research Support|CyanVac: Grant/Research Support|GSK: Advisor/Consultant|GSK: Travel support|Janssen: Grant/Research Support|Moderna: Advisor/Consultant|Moderna: Grant/Research Support|Pfizer: Advisor/Consultant|Pfizer: Grant/Research Support|Sanofi Pasteur: Advisor/Consultant|Sanofi Pasteur: Travel support|VaxCo: Grant/Research Support **Angela R. Branche, MD**, Cyanvac: Grant/Research Support|GSK: Advisor/Consultant|Merck: Grant/Research Support|Moderna: Grant/Research Support|Moderna: Honoraria|Novavax: Advisor/Consultant|Pfizer: Grant/Research Support|Sanofi: Honoraria|VaxCo: Grant/Research Support **Edward E. Walsh, MD**, Enanta: Advisor/Consultant|Enanta: Honoraria|GSK: Advisor/Consultant|Janssen: Advisor/Consultant|Merck: Advisor/Consultant|Merck: Grant/Research Support|Pfizer: Advisor/Consultant|Pfizer: Grant/Research Support

